# Advances in research of biological functions of Isthmin-1

**DOI:** 10.1007/s12079-023-00732-3

**Published:** 2023-03-30

**Authors:** Li Menghuan, Yang Yang, Ma Qianhe, Zhang Na, Cao Shicheng, Chang Bo, Y. I. XueJie

**Affiliations:** 1 School of Sports and Human Sciences, Shenyang Sport University, No. 36 Qiangsong East Road, Sujiatun District, Shenyang, 110102 China; 2 School of Sports and Human Sciences, Shanghai Sport University, Shanghai, 200438 China; 3 School of Physical Education, Liaoning Normal University, Dalian, 116029 China; 4 Exercise and Health Research Center/Department of Kinesiology, Shenyang Sport University, No.36 Qiangsong East Road, Sujiatun District, Shenyang, 110115 Liaoning Province China; 5 Department of Sports Medicine, China Medical University, Shenyang, China

**Keywords:** Isthmin-1, Growth and development, Glycolipid metabolism, Protein metabolism, Apoptosis, Anti-angiogenesis, Immunity

## Abstract

Isthmin-1 (ISM1) was initially thought to be a brain secretory factor, but with the development of technical means of research and the refinement of animal models, numerous studies have shown that this molecule is expressed in multiple tissues, suggesting that it may have multiple biological functions. As a factor that regulates growth and development, ISM1 is expressed in different animals with spatial and temporal variability and can coordinate the normal development of multiple organs. Recent studies have found that under the dependence of a non-insulin pathway, ISM1 can lower blood glucose, inhibit insulin-regulated lipid synthesis, promote protein synthesis, and affect the body's glucolipid and protein metabolism. In addition, ISM1 plays an important role in cancer development by promoting apoptosis and anti-angiogenesis, and by regulating multiple inflammatory pathways to influence the body's immune response. The purpose of this paper is to summarize relevant research results from recent years and to describe the key features of the biological functions of ISM1. We aimed to provide a theoretical basis for the study of ISM1 related diseases, and potential therapeutic strategies.

**Graphical abstract:**

The main biological functions of ISM1. Current studies on the biological functions of ISM1 focus on growth and development, metabolism, and anticancer treatment. During embryonic development, ISM1 is dynamically expressed in the zebrafish, African clawed frog, chick, mouse, and human, is associated with craniofacial malformations, abnormal heart localization, and hematopoietic dysfunction. ISM1 plays an important role in regulating glucose metabolism, lipid metabolism, and protein metabolism in the body. ISM1 affects cancer development by regulating cellular autophagy, angiogenesis, and the immune microenvironment.

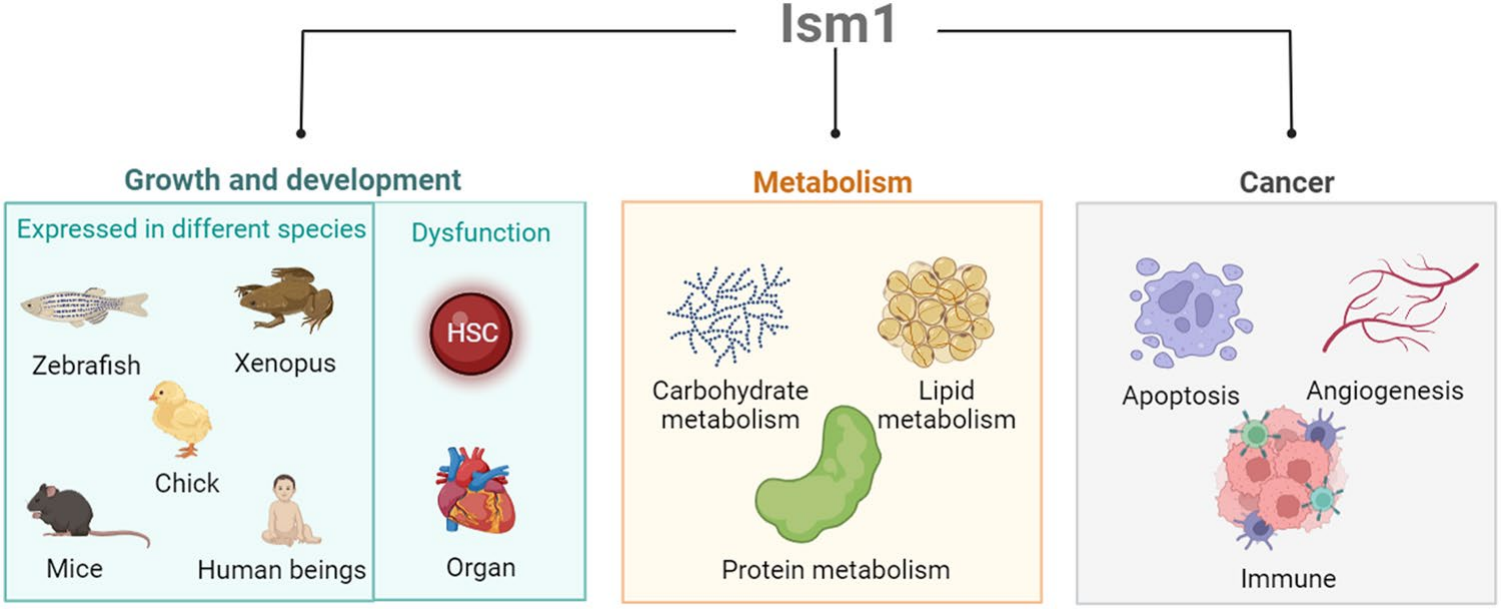

## Foreword

Isthmin-1 (ISM1) is a new protein family member that was first identified by Pera et al. (Pera et al. 2002) in 2002 when screening for secreted proteins in *Xenopus laveis* (clawed frog) embryos. It was named for its significant expression at the midbrain-hindbrain boundary (MHB), or the isthmus of the brain (Pera et al. 2002). Studies have shown that ISM1 is present in the genomes of many species of chordates and is dynamically expressed during embryonic development to regulate growth and development (Osório et al. 2014; Pera et al. 2002). In adulthood, ISM1 is expressed in a variety of tissues and organs and is organ-tissue specific (Osório et al. 2014). It is noteworthy that ISM1 is expressed at different sites in different animals, suggesting that ISM1 may not mediate the same functions in different organs of different animals.

In September 2021, Jiang Zewen et al. (Jiang et al. 2021) discovered that ISM1 is synthesized in mature adipocytes and that it promotes glucose uptake in a non-insulin-dependent pathway that inhibits hepatic fat synthesis and promotes hepatic protein synthesis. These findings are expected to provide new targets for the prevention and treatment of diseases associated with dysregulated glucolipid metabolism, such as type 2 diabetes mellitus and non-alcoholic fatty liver disease (NAFLD) among others.

In addition, it has been reported that ISM1 binds to important molecules like αvβ5 integrin and glucose regulatory protein 78 (GRP78). It also plays a role in regulating apoptosis and anti-angiogenesis, and acts on pathways that include transforming growth factor β (TGF-β), interleukin-6/Janus kinase/signal transducer and activator of transcription 3 (IL-6/JAK/STAT3), interferon γ (IFN-γ), tumor necrosis factor-α/nuclear factor κB (TNF-α/NF-κB), IL-2/STAT5, and other pathways involved in regulating the occurrence and development of cancer(M. Chen et al. 2014; Yuan et al. 2012). In this article, we searched the relevant literature of ISM1 in growth and development, glycolipid metabolism, anti-cancer and other functions from 2002 to 2022 through PubMed, China Knowledge Network, Web of Science, Google Scholar and Baidu Academic databases and sorted them out. A review of the multiple biological functions of ISM1 was conducted. Herein, the mechanism of action of ISM1 and possible future research directions are described, with the aim of providing a theoretical basis for subsequent related research and a reference for the treatment of related diseases.

## Overview of Isthmin-1

The *ISM* gene was originally screened and detected in African *Xenopus* embryos. It is considered to be a novel member of the MHB-secreted protein family because it is highly expressed in MHB and its carboxy-terminal functional region has no known features detected in the protein structure domain database (Pera et al. 2002). The *ISM* genome sequence is over 80 kb in size and contains 6 exons and 5 introns (Osório et al. 2014; Pera et al. 2002). Further studies have shown that ISM in the mammalian genome can be divided into *ISM1* and *ISM2*/*Tail* (Pera et al. 2002; Valle-Rios et al. 2014). ISM1 is present in many vertebrate genomes, including birds, fish, amphibians, and mammals (Osório et al. 2014; Pera et al. 2002; Valle-Rios et al. 2014). Structurally, the *ISM1* gene encode three α-helices and two β-folds and is located in chicken chromosome 3, mouse chromosome 2, and human chromosome 20 (Osório et al. 2014; Valle-Rios et al. 2014).

Mouse ISM1 encodes a protein of 461 amino acids and human ISM1 encodes a protein of 499 amino acids, and there is a high degree of homology between mouse and frog, chick, and human protein sequences, sharing 78%, 80%, and 93% identity, respectively (Osório et al. 2014). The molecular weight of the ISM1 protein was found to be about 60 KDa in frogs, 50 KDa in mice, and 70 KDa in humans (Osório et al. 2014; Pera et al. 2002; Valle-Rios et al. 2014). Since the molecular weight of ISM1 varies in different animals, it is suggested that there might be post-translational modifications of ISM1. Furthermore, bioinformatic analysis has revealed the presence of multiple protein sequences in ISM1, including the N-terminal signal peptide, the centrally located thrombospondin type 1 repeat (TSR1), and the adhesion-related structural domains of MUC4 and other proteins (AMOP) at the C-terminus (Osório et al. 2014; Pera et al. 2002; Rossi et al. 2004).

Studies have shown that the TSR1 structural domain is about 60 amino acids in length and has an important role in regulating cell adhesion and migration, wound healing, and angiogenesis (Tucker 2004). The AMOP domain, which is about 160 amino acids in length, is only found in extracellular proteins involved in cell adhesion and is closely related to pancreatic cancer angiogenesis and metastasis (Ciccarelli et al. 2002; Tang et al. 2016). Osório et al. (Osório, et al. 2014) confirmed that mouse ISM1 is N-glycosylated at both Asn39 and Asn282 through mutational analysis of these residues. They also revealed that these are no longer sensitive to inhibitors of N-linked glycosylation after mutation, as predicted by sequence analysis of the adult mouse ISM1 protein (Osório et al. 2019). Subsequently, further studies revealed that ISM1 expression was significantly reduced after N-glycosylation inhibitor treatment, indicating the importance of N-glycosylation of *ISM1* for its secretion (Osório et al. 2019). Yoshimoto et al. (Yoshimoto, Katayama, Suzuki, Dohmae and Simizu 2021) found that the TSR1 structural domains, Trp223 and Trp226, of human ISM1 are glycosylated by C-mannitol, which facilitates endoplasmic reticulum-to-Golgi transport and protein secretion, while the Asn39 and Asn285 sites of ISM1 N-glycosylation rescues secretory function when mannitol glycosylation is inhibited. Interestingly, because the TSR1 structural domain of human ISM1 is highly conserved, C-mannosylation of ISM1 may also be present in other animals. The role of post-translational modifications of ISM1 is less studied and may become a focus of future studies.

The two structural domains of ISM1 are highly conserved among species and have high homology, such as 98% between mouse and human TSR1 and 99% between their AMOP domains. Furthermore, there is 87% and 88% homology between mouse and zebrafish TSR1 and that between mouse and African clawed frog respectively, and 85% and 81% homology between their respective AMOP domains (Xiang et al. 2011). Fibroblast growth factor 8 (Fgf-8) has previously shown to be an important signaling factor mediating MHB activity in embryos of several species (Harada et al. 2016). It has been confirmed that ISM1 and Fgf-8 genes are expressed at very similar sites, with partially overlapping or co-expressing expression domains at the anterior margin of the neural plate, MHB, gill arch, posterior mesencephalon, and ear capsule in African clawed frogs (Pera et al. 2002). The expression domains of ISM1 in zebrafish embryos include the dorsal edge of the embryo, the dorsal hypoderm, the posterior medial part of the mesoderm, the MHB, and the notochord; these ISM1 expression locations are very similar to those of Fgf-8 (Kesavan et al. 2021). This also reaffirms the conserved expression of ISM1 across species and the possible important role of ISM1 in neurological development during the embryonic stage.

## Biological function of ISM1

### ISM1 in growth and development

Current reports on the role of ISM1 focus on its dynamic expression during different periods of embryonic development and the fact that it affects the embryonic body axis, organs, craniofacial morphology, and hematopoietic cell formation.

#### Dynamic expression of ISM1

Several studies have shown that ISM1 is expressed early in the embryonic development of frogs and zebrafish (Pera et al. 2002; Xiang et al. 2011). Pera et al. (Pera et al. 2002) observed that ISM1 was expressed in several sites in frog embryos, including the ventral ectodermal lip, proximal mesoderm, neural folds, notochord, MHB, isthmus, and hind mesencephalon.Additionally, Ism1 is expressed in the branchial arches. A correlation study of the timing and location of ISM1 expression in zebrafish found that it was expressed in the MHB and hind trunk regions during the late prometaphase/early segmentation stage and thereafter only in the tail bud region and notochord; the expression level in the notochord increased at 22 h post fertilization (hpf), decreased in the tail bud at 30 hpf, decreased in the notochord at 48 hpf, and disappeared at 72 hpf (Kesavan et al. 2021). Interestingly, deletion of ISM1 did not affect embryonic morphological changes during early development of zebrafish embryos, which may be triggered by genetic compensation phenomena through mutations in other genes (El-Brolosy et al. 2019; Kesavan et al. 2021).

The expression of ISM1 in chicks is dynamic. In 10-somites embryos, ISM1 expression is spatially restricted to the anterior region (Osório et al. 2014). At 21–22 somites, ISM1 transcripts are strongly expressed in the epithelium of the ventral ear sac and begin to be weakly expressed in the neural tube of the anterior trunk region (Osório et al. 2014). In embryos of 31–33 somites, ISM1 is expressed in the epithelium of the optic cup and ventral midbrain, as well as the ear vesicle, the neural tube, the dermatomyotome, and the lateral plate mesoderm (Osório et al. 2014). The absence of ISM1 expression in the MHB and precursor mesoderm or somites of chick embryos may be related to species differences and varying differentiation mechanisms of somites and dermomyotomes (Osório et al. 2014).

ISM1 also starts to be expressed early in mouse embryonic development, and the regions where ISM1 transcripts exist differ depending on the stage of development; for example, low levels of ISM1 can be observed at embryonic day 6.75, ISM1 transcripts are expressed in the anterior mesoderm at embryonic day 7.5, and the expression level gradually increases as ISM1 transcripts become present in the somites, MHB, anterior somites, anterior and lateral plate mesoderm, ear vesicle, and neural tube (Osório et al. 2014). The expression of ISM1 in the dorsal aspect of the trunk neural tube in mice starts at 5–6 somites and adapts in a gradient according to he anterior–posterior axis of the embryo, suggesting that ISM1 may be associated with spine formation (Osório, et al. 2014). Analysis against human gene expression databases revealed that skin, mucosal tissue, and lymphoid tissue cells express ISM1 and have barrier functions; however, the human nervous system has low levels of ISM1 expression (Valle-Rios, et al. 2014). The identification of ISM1-expressing lymphocytes revealed that human and mouse CD4^+^ T cells express ISM1 upon activation, which suggests that ISM1 may be associated with the body's innate and adaptive immunity (Valle-Rios, et al. 2014). Furthermore, although ISMI is present in the genomes of a wide range of vertebrates, its expression in mammals differs from that of other animals. ISM1 is virtually absent from the mammalian nervous system, but it is present in CD4^+^ T cells of peripheral blood. Thus, ISM1 is expressed in organs of multiple animals at different times and may affect the growth and development of the sites in which it is expressed.

Studies have shown that ISM1 is directly and indirectly controlled by β-catenin. Injection of the nodal signaling gene, *sqt*, and the homologous frame transcription factor, bozozok (*boz*), mRNA during zebrafish gastrula embryogenesis resulted in ectopic expression of ISM1 in the embryonic marginal region, as well as upregulation of ISM1 expression, suggesting that both genes may be upstream of ISM1 (Kesavan et al. 2021). Notably, *sqt* and *boz* upregulate ISM1 expression even when β-catenin is blocked, which, combined with previous studies reporting that β-catenin is a transient signaling molecule, suggests that the Wnt/β-catenin pathway may not be required for ISM1 activation (Kesavan et al. 2021). However, in contrast to these early observations, late ISM1 expression appears to be independent of nodal signaling (Kesavan et al. 2021), suggesting that ISM1 expression is temporally controlled by a different signaling system.

#### ISM1 and the growth and development process

Orofacial cleft is a common congenital malformation with a complex etiology that is influenced by both genetic and environmental factors (Martinelli, Palmieri, Carinci, and Scapoli 2020; Nasreddine, El Hajj, and Ghassibe-Sabbagh 2021). FGFs, Sprouty Homolog 1 (*Spry1*), and *Spry2* in Fgf-8 are also involved in craniofacial development and associated with cleft lip or palate (Conte et al. 2016; Porntaveetus et al. 2010; Reynolds et al. 2020). Since the ISM1 expression pattern is similar to and belongs to the same expression group as Fgf-8, it is expressed in MHB, ear substrate, maxilla, mandible, periorbital, and other sites important for craniofacial development,  including branchial arch 1 (BA1) which gives rise to the maxilla, the structure that is associated with cleft lip and/or palate (Osório et al. 2014; Pera et al. 2002). Craniofacial defects occur in cases with heterozygous deletions of ISM1; therefore, Lansdon et al. (Lansdon et al. 2018) identified ISM1 as an orofacial clefting candidate for study and concluded that it was haploinsufficient. Inhibition of ISM1 in African clawed frog embryos showed shortened embryonic body axes, missing or defective eyes, abnormal tail, and craniofacial malformations including median facial clefts analogous to those found in humans, where increasing inhibition caused whole embryo abnormalities or even headlessness (Lansdon et al. 2018). Furthermore, the expression of LIM homeobox protein 8 (*Lhx8*), which is associated with craniofacial morphology, decreased with the knockdown of ISM1 (Lansdon et al. 2018). This suggests that ISM1 is involved in craniofacial development (Table 1, Fig. 4).

**Table 1:** Expression and biological functions of ISM1 in different sites

References	ISM1 expression site	Mode	Function
Osório (Osório et al. 2019)	Chick embryo mesoderm, endoderm	ISM1 suppresses the NODAL signal	Embryonic left–right asymmetry and abnormal heart positioning
Lansdon (Lansdon et al. 2018)	Mesoderm, midbrain-hindbrain boundary (MHB), primitive mouth region, branchial arches, cranial neural crest cells of Xenopus laevis embryos.	Inhibition of ISM1	Embryonic body axis shortening, craniofacial malformation, or even headless
Berrun (Berrun et al. 2018)	Zebrafish embryonic stromal stem cell line, embryonic caudal hematopoietic stromal tissue cell line, and zebrafish renal stromal cell line	Inhibition of ISM1	Decreased HSPC, affecting hematopoiesis
Rivera (Rivera-Torruco et al. 2022)	Mouse lung		Affecting hematopoiesis
Jiang Z (Jiang et al. 2021)	Mouse adipocytes	ISM1-mTORC2/PI3K-AKT-mTORC1	Increase glucose intake
Jiang Z (Jiang et al. 2021)	Mouse hepatocytes	ISM1-pS6^S235/S236^	Promotes protein synthesis
Meng Z(Zhao et al. 2022)	Mouse skeletal muscle cells	ISM1-pS6^S235/S236^	Promotes protein synthesis
Jiang Z (Jiang et al. 2021)	Mouse hepatocytes	ISM1-cSrebp1c-ACC, FAS, SCD	Inhibit adipose de novo synthesis
Wu Y (Wu et al. 2021)	Human and mouse CD4^+^ T	Enrichment Analysis	Associated with multiple classical inflammatory pathways, mediates cancer immunity

**Fig. 1:**
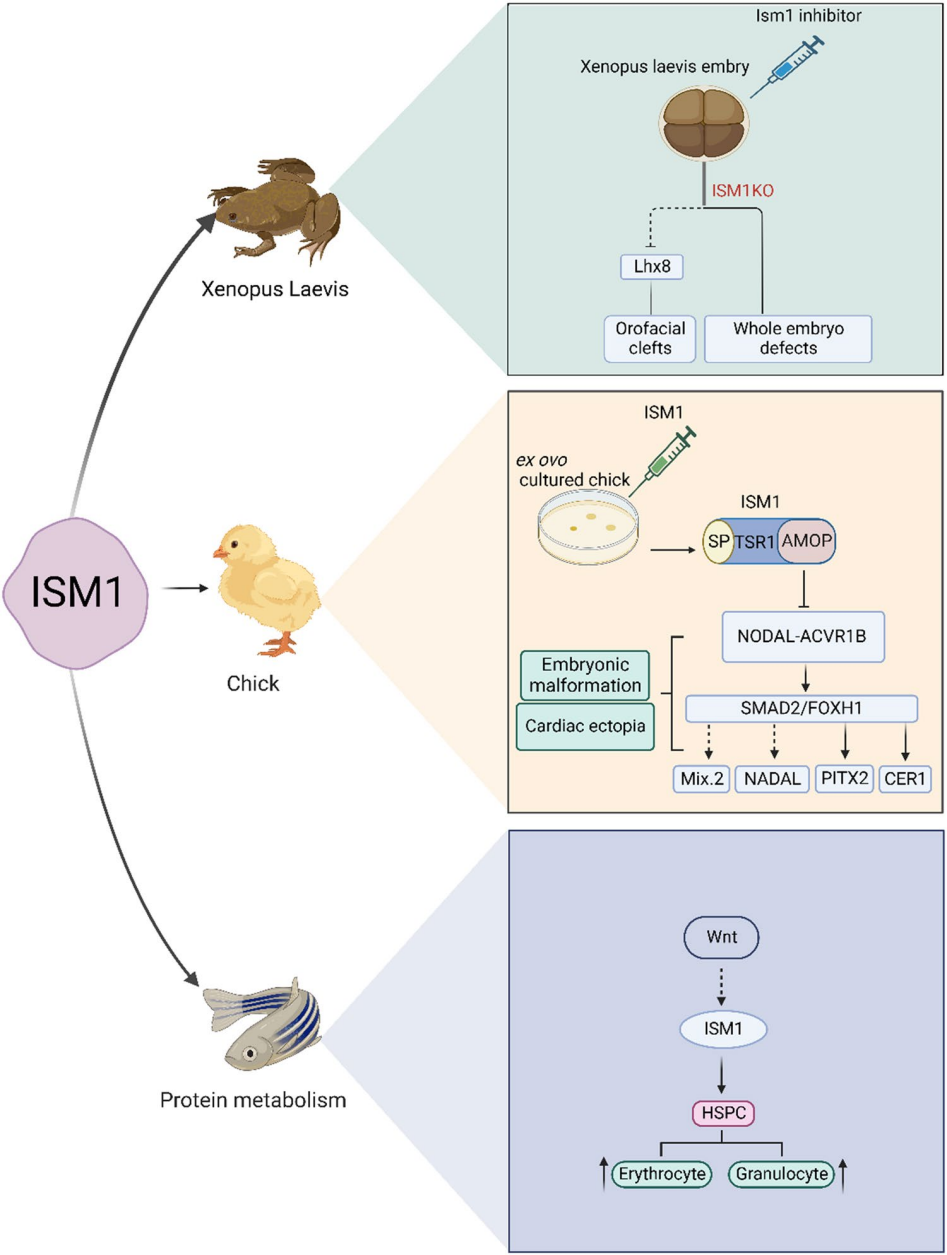
Effect of ISM1 on organ morphology and hematopoiesis during growth and development

﻿ISM1 deficiency in Xenopus laevis embryos may cause craniofacial malformations via Lhx8, causing whole-embryo abnormalities as the degree of inhibition increases. In chick embryos, ISM1 inhibits NODAL-SMAD2 and downstream targets, leading to left–right asymmetry and abnormal heart localization in chick embryos. Wnt is both an important factor mediating HSC production and an upstream signal for ISM1, and ISM1 knockdown leads to reduced HSPC production and fewer mature erythrocytes and bone marrow cells, so Wnt may affect hematopoiesis through ISM1 cell production. (Paired-liked homeodomain transcription factor 2, PITX2)、CER1 (Cerberus 1).

The TGF-β superfamily includes several subfamilies of TGF-β, NODAL, growth differentiation factors (GDF), and activating and bone morphogenetic proteins (BMP), which play important roles in embryonic development, body immunity, and cancer (Hayes et al. 2021; Magro-Lopez and Muñoz-Fernández 2021; Stuelten and Zhang 2021). ISM1 contains a TSR1 structural domain, which typically mediates TGF-β family signaling, suggesting that ISM1 may be involved in regulating TGF-β signaling (Adams and Lawler 2011; Rossi et al. 2004). Osório et al. (Osório et al. 2019) found that ISM1 caused a significant decrease in phosphorylation of the ligand SMAD2 of NODAL by examining the effect of ISM1 on the main ligands of the TGF-β family, while the effect on the expression of several other ligands was not significant. Interestingly, it was shown that the loss of inhibitory function of ISM1 on NODAL in the absence of the AMOP structural domain was not significantly affected when the TSR1 structural domain was deleted, suggesting that the effect of ISM1 on NODAL may not be dependent on TSR1, but on the AMOP structural domain (Adams and Lawler 2011; Osório et al. 2019; Pera et al. 2002). In addition, NODAL is important for the development of the embryonic mesoderm and endoderm, as well as for the formation of the anterior–posterior and left–right body axes (Schier 2009). *ISM1* is highly expressed in the anterior mesoderm of chick and mouse embryos, and mesoderm and endoderm formation is associated with NODAL signaling, which is consistent with the fact that *ISM1* is thought to be a regulatory gene for NODAL signaling in zebrafish embryos (Bennett et al. 2007; Montague and Schier 2017)*. *In vitro, the AMOP structural domain of ISM1 inhibits NODAL during development by interacting with the activin A receptor type 1B ligand, resulting in left–right asymmetry and abnormal heart positioning in chick embryos. (Table 1, Fig. 1).

In spinal animals, hematopoietic stem cells (HSC) are constantly self-renewing and differentiating to maintain blood homeostasis throughout development (Montazersaheb et al. 2022). HSCs differentiate into populations of progenitor cells of various types of blood cells, which together maintain the body's blood cell population (Manz et al. 2002; Weinreb, Rodriguez-Fraticelli, Camargo, & Klein, 2020). The zebrafish is a perfect model for studying the biology of hematopoietic stem and progenitor cells (HSPCs) due to its similarity of circulatory and hematopoietic systems to humans and its transparent coloration (Carroll and North 2014; Gore, Pillay, Venero Galanternik, and Weinstein 2018). Berrun et al. (Berrun, Harris, & Stachura 2018) showed that ISM1 expression levels were high in all three cell lines (zebrafish embryonic stromal stem cell line, embryonic caudal hematopoietic stromal tissue cell line, and zebrafish renal stromal cell line), supporting the idea of different time points of hematopoiesis in zebrafish, and that knockdown of ISM1 results in a decrease in bone marrow cells, such as erythrocytes, neutrophils, and macrophages, in the organism; furthermore, ISM1 deletion resulted in a decrease in HSPC production. Previous studies have shown that Wnt, an important factor mediating HSC production, can in turn regulate ISM1 expression, which may explain the involvement of ISM1 in hematopoietic cell formation during zebrafish development (Campbell et al. 2015; Stachura et al. 2009; Wolf et al. 2017). In mice, the lungs are one of the active hematopoietic organs (Lefrançais et al. 2017). ISM1, expressed mainly in lung NK and NKT-like cells in mice (Osório et al. 2014; Valle-Rios et al. 2014). Rivera et al. (Rivera-Torruco et al. 2022) recently showed that mice ISM1^+^ cells have a progenitor phenotype associated with endothelial cells (EC), mesenchymal cells, and hematopoietic cells in the lungs, and that ISM1^+^ LSK cells may represent a subset of hematopoietic progenitors. These results suggest that ISM1 is closely associated with the hematopoietic function (Fig. 1) In addition, recent studies have shown that gene enrichment analysis of *ISM1* is associated with TGF-β signaling, which has an important role in embryonic development, hematopoiesis, and spinal cord disease (Bataller et al. 2019; Wu et al. 2021). However, whether *ISM1* is involved in hematopoiesis via TGF-β needs further investigation.

ISM1 deficiency in zebrafish embryos may cause craniofacial malformations via Lhx8, causing whole-embryo abnormalities as the degree of inhibition increases. In chick embryos, ISM1 inhibits NODAL-SMAD2 and downstream targets, leading to left–right asymmetry and abnormal heart localization in chick embryos. Wnt is both an important factor mediating HSC production and an upstream signal for ISM1, and ISM1 knockdown leads to reduced HSPC production and fewer mature erythrocytes and bone marrow cells, so Wnt may affect hematopoiesis through ISM1 cell production. (Paired-liked homeodomain transcription factor 2, PITX2)、CER1 (Cerberus 1).

### ISM1 and metabolism

Adipose tissue, the liver, and skeletal muscle play a vital role in maintaining systemic energy homeostasis (Korenblat et al. 2008). In the state of obesity, excessive energy intake leads to subcutaneous fat spillage, which causes ectopic lipid accumulation in skeletal muscle, liver, and other tissues, decreased glucose tolerance, and inflammatory reactions, resulting in a variety of metabolic diseases. Insulin is the only hypoglycemic hormone in the body, and it not only promotes glucose uptake, but also promotes lipid synthesis, which may even lead to obesity or the development of alcoholic fatty liver disease in the case of hyperinsulinemia (Kolb et al. 2020). Therefore, finding cytokines that can both increase glucose uptake and inhibit lipogenesis may be a crucial direction for current glucose uptake research. Recent studies have defined ISM1 as an adipokine that has important roles in promoting glucose uptake, inhibiting lipogenesis, and stimulating protein synthesis.

#### ISM1 and glucose metabolism

ISM1 positively correlates with obesity in human and mouse adipocytes, and in the plasma of females (Jiang et al. 2021). However, circulating ISM1 levels were lower in the middle-aged overweight population with type 2 diabetes than in the non-type-2-diabetic overweight group; this suggests that elevated ISM1 may reduce the risk of developing diabetes (J. Wang et al. 2022).

In 2021, by sequencing RNA from brown and white mature adipocytes in mice and performing bioinformatics analysis, Jiang et al. (Jiang et al. 2021) found that ISM1 was highly expressed in mature adipocytes, especially brown adipocytes, suggesting that ISM1 may be closely related to the function of mature adipose tissue. This study used recombinant ISM1 or insulin to treat human SGBS adipocytes, primary mouse adipocytes, 3T3-L1 adipocytes, and human skeletal muscle cells, which are closely related to glucose metabolism (Jiang et al. 2021). The study found that ISM1 mediated glucose uptake in a variety of cells in a non-insulin-dependent pathway, and that ISM1-mediated glucose uptake capacity differed between different cell types and species, which may be related to ISM1-dependent cell type-specific receptors or glucose transporter proteins (Jiang et al. 2021). Glucose transporter 4 (GLUT4) is a glucose transporter protein regulated mainly by adenosine triphosphate (ATP) or insulin in adipose and skeletal muscle cells (Fazakerley et al. 2022). In vitro experiments have demonstrated that ISM1 promotes GLUT4 translocation from the cytoplasm to the plasma membrane, while endogenous phosphorylation of the energy metabolism factor AKT^S473^ increases glucose uptake (Jiang et al. 2021). ISM1 was able to induce pAKT^S473^ phosphorylation levels in a variety of mature adipocytes and human primary skeletal muscle cells, and treatment with phosphatidylinositol 3-kinase (PI3K) inhibitors revealed that ISM1-induced glucose uptake was completely blocked, suggesting that that ISM1 requires PI3K to induce glucose uptake in adipocytes (Jiang et al. 2021).

**Fig. 2:**
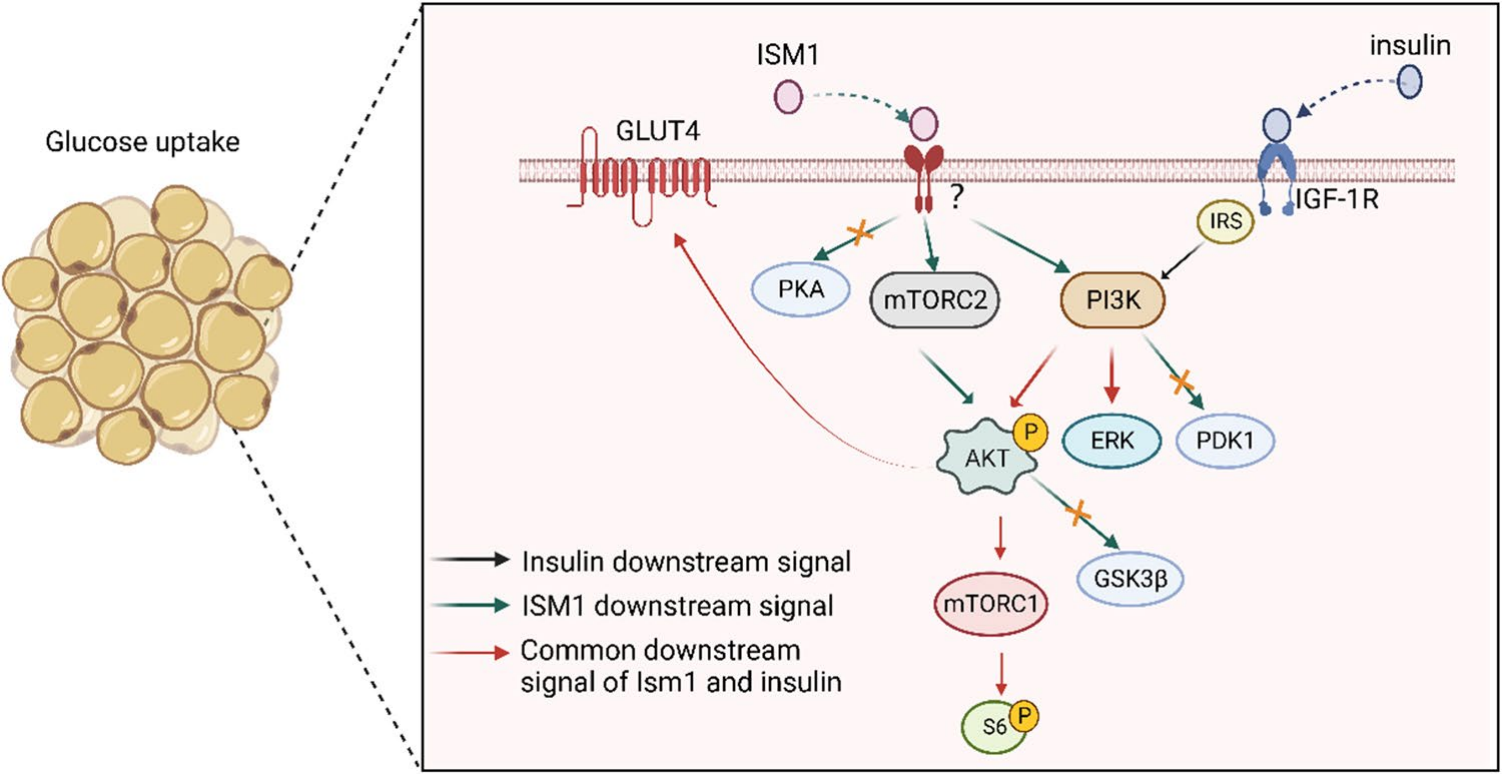
Diagram of the mechanism of ISM1 regulation of glucose metabolism

The mammalian target of rapamycin (mTOR) and its complexes mTORC1 and mTORC2 are activated by insulin, which in turn regulates insulin sensitivity (Destefano and Jacinto 2013). Treatment of cells with mTORC1, 2 inhibitors followed by stimulation with ISM1 or insulin revealed that mTORC2 inhibitors blocked ISM1induced AKT signaling, while mTORC1 inhibitors did not, suggesting that ISM1 may be involved in the induction of PI3K-AKT signaling pathway and glucose uptake through mTORC2 (Jiang et al. 2021). However, when both mTOR inhibitors were present, ISM1 induced a complete loss of phosphorylation of S6^S235/S236^ and unchanged AKT phosphorylation levels, suggesting that ISM1 induces activation of downstream SD ^S235/S236^ via mTOR1 (Jiang, et al. 2021). Notably, although ISM1 and insulin have similar regulatory effects on glucose, ISM1 does not act on the insulin receptor, but rather activates the PI3K-AKT pathway by binding to a unique receptor to promote glucose uptake by adipocytes. (Table 1, Fig. 2).

In metabolism, ISM1 secreted by adipocytes acts in an autocrine manner to regulate glucose uptake via mTORC2-PI3K-AKT in conjunction with insulin-IR/IGF-1R-PI3K-AKT. ISM1 is able to cause ERK phosphorylation but has no significant effect on other signaling pathways such as PKA, PDK1 or GSK3β.

#### ISM1 and lipid and protein metabolism

**Fig. 3:**
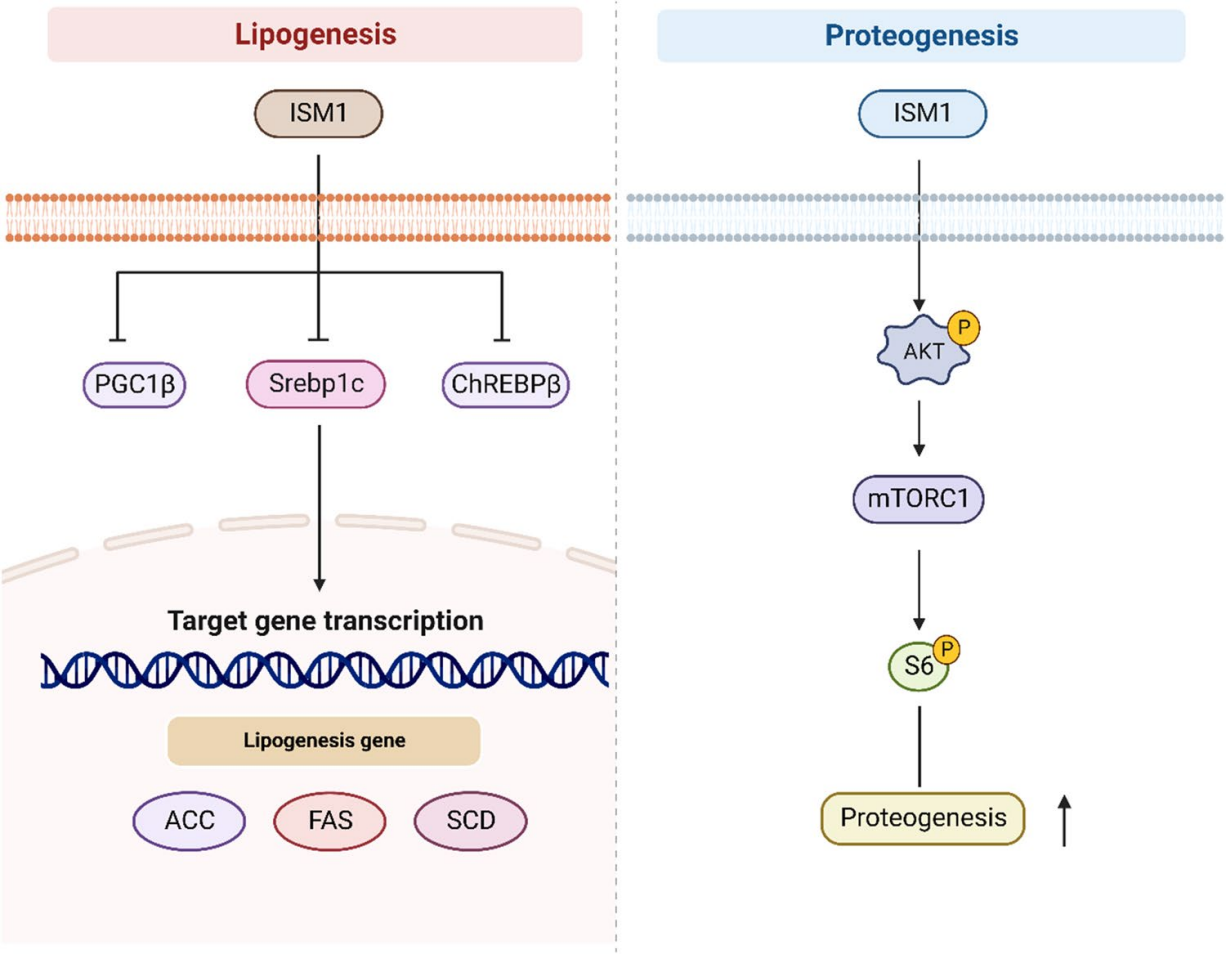
Diagram of the mechanism of ISM1 regulation of lipid metabolism and protein metabolism

In a high-fat diet-induced obesity and nonalcoholic fatty liver mouse model, injection of recombinant ISM1 can effectively reverse hepatic steatosis, which may provide a new direction for clinical treatment of metabolic diseases (Jiang et al. 2021). ISM1 is similar to insulin in function and downstream signaling pathways, thus ISM1 may have similar roles to insulin in regulating glucose uptake and lipogenesis. Interestingly, ISM1 inhibits insulin-induced expression of the pro-lipid synthesis factor sterol regulatory element binding protein-1 (cSrebp1c) and its target genes acetyl CoA carboxylase (ACC), fatty acid synthase (FAS), and low density lipoprotein receptor (LDLR) in a dose-dependent manner, thereby attenuating insulin-induced lipid de novo lipogenesis(Jiang, et al. 2021). Since ISM1 also represses the expression of carbohydrate response element binding protein (ChREBPβ) and peroxisome proliferator-activated receptor γ coactivator 1β (PGC1β) (Jiang, et al. 2021), ISM1 may regulate lipogenesis through multiple pathways. Unfortunately, this experiment only targeted mature adipocytes for detection of ISM1 and did not account for the lipolytic effect on adipocytes at different growth stages. Thus, it is worth further exploring whether ISM1 can directly affect adipocytes at different growth stages in the future (Fig. 3).

﻿﻿﻿In﻿ hepatocytes ISM1 inhibits the expression of Srebp1c and its target genes and affects lipid de novo synthesis. In hepatocytes and skeletal muscle cells, it promotes protein synthesis via AKT-mTORC1-S6.

In hepatocytes, ISM1 induces pS6^S235/S236^ overactivation, and the combined action of ISM1 and insulin maintains pS6^S235/S236^ and increases protein synthesis 2.9-fold (Jiang et al. 2021). ISM1 inhibits lipogenesis by switching the cellular anabolic state to a protein synthesis state (Jiang et al. 2021). Skeletal muscle not only controls whole-body energy expenditure, but also serves as a reservoir of protein and is highly sensitive to protein anabolism and catabolism. Recent studies have shown that ISM1 can act directly on skeletal muscle cells to induce protein synthesis via pAKT- pS6^S235/S236^ (Zhao et al. 2022). Skeletal muscle fibers became smaller, protein degradation increased, muscle strength decreased, and muscle atrophy-related FOXO1 target gene levels increased with *ISM1* knockdown (Zhao et al. 2022). This suggests that ISM1 may affect the contractile function of skeletal muscle by regulating its protein synthesis. In addition, muscle function decreases in the obese state, but further studies on the effect of ISM1 on muscle function in the obese state are needed (Fig. 3).

Aging refers to the gradual decline in body functions with age throughout the life cycle (Flatt 2012). The aging process is usually accompanied by dysregulation of glucose and lipid metabolism (Barbé-Tuana et al. 2020). In *Nothobranchius guentheri,* a fish, ISM1 expression levels decreased with age in the liver, muscle, and serum. Control fish and fish with recombinant ISM1 fed with a normal diet showed that recombinant ISM1 prolonged lifespan and reduced the accumulation of aging markers such as liver lipofuscin, reactive oxygen species, and protein oxidation and lipid peroxidation in fish muscle (C. Li et al. 2022). Unfortunately, this study did not identify the molecular mechanisms by which ISM1 affects aging. Previous studies have shown that aging is associated with NF-κB, PI3K/Akt/mTOR, Wnt/β-catenin, and AMPK signaling pathways (Y. Liu, Weng, Gao, and Liu 2019). However, ISM1 is a downstream factor of the Wnt/β-catenin pathway, and gene enrichment experiments have shown that it is highly associated with PI3K/Akt/mTOR and NF-κB (Jiang et al. 2021; Wu et al. 2021). Therefore, ISM1 may act via these pathways. In addition, oxidative stress and lipid peroxidation are associated with obesity, diabetes, and NAFLD, and ISM1 may improve metabolic diseases by reducing oxidative stress and lipid peroxidation (Tchkonia et al. 2010). GRP78 which is present on the cell surface is a high-affinity receptor for ISM1 (M. Chen et al. 2014). Whether ISM1 improves inflammation, metabolic disorders, and endoplasmic reticulum stress in diet-induced obesity through GRP78, and whether ISM1 plays a role in metabolic diseases such as obesity, type 2 diabetes, and alcoholic fatty liver and the mechanisms by which these are regulated are still unclear. Exploring this will be important in directing future research (Luo et al. 2022; Zhu et al. 2013).

### ISM1 and cancer

There will be about 19.3 million new cases and 10 million deaths due to cancer globally in 2020, and cancer incidence and mortality rates continue to climb year by year (Sung et al. 2021). Apoptosis, angiogenesis, inflammation, and other factors can affect the development of cancer (Carneiro and El-Deiry 2020; Kaczanowski 2016; Senga and Grose 2021). In recent years, ISM1 has been found to play an important role in anti-angiogenesis, regulation of immune response, and promotion of apoptosis; thus, it has been found to have great anti-cancer potential.

#### Apoptosis

Apoptosis is an important form of programmed cell death which plays an important role in maintaining tissue and organ development, eliminating abnormal malignant cells, and regulating the immune and internal homeostasis of the body (Cao and Tait 2018). However, abnormal changes in the process of apoptosis, such as gene mutations and occurrence of apoptosis, may lead to the development of cancer (Wong 2011).

**Fig. 4:**
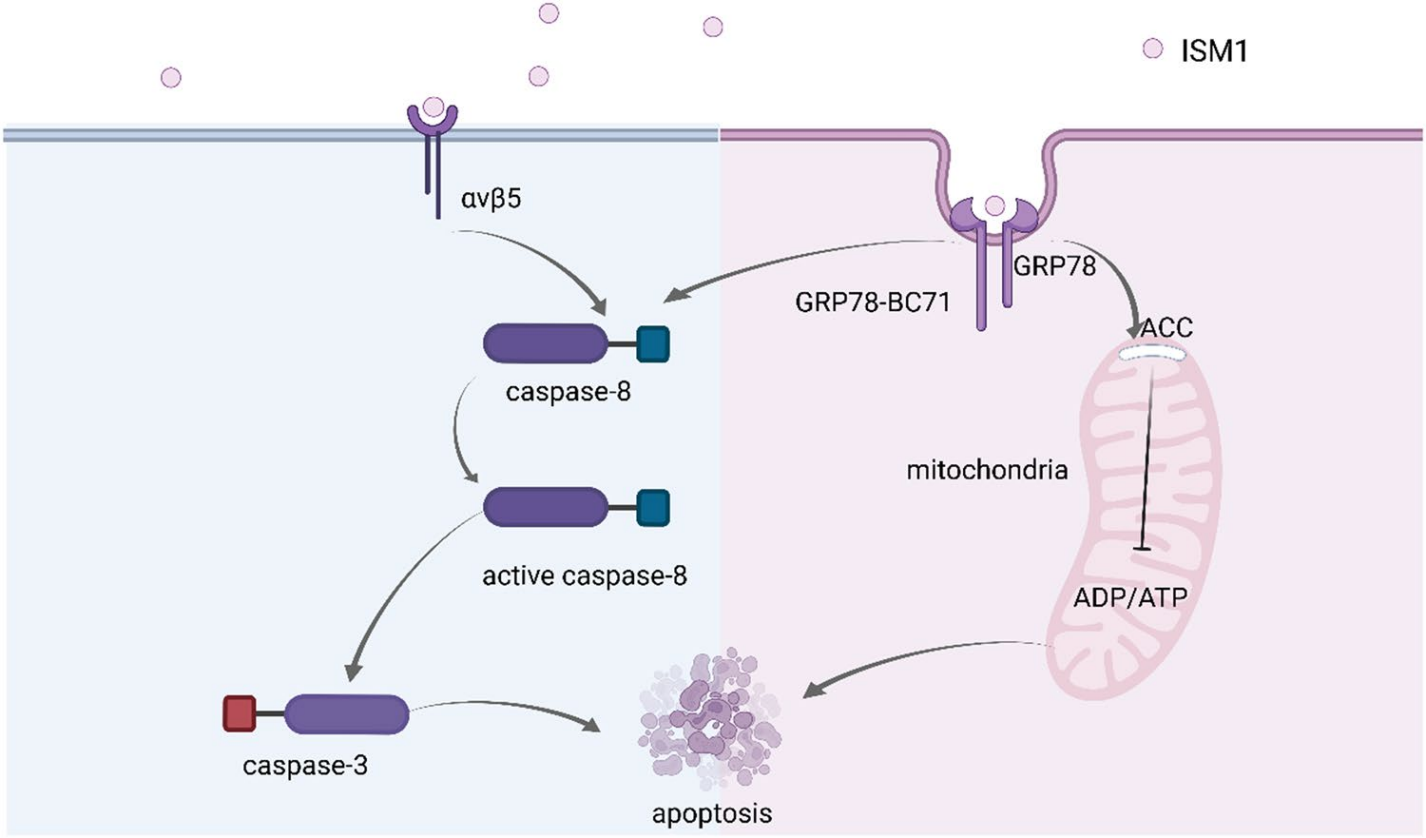
The mechanism of ISM1 regulation of apoptosis.

The role of caspases is crucial in intracellular apoptosis (Hou et al. 2022; Park et al. 2022; Sevimli, Bayram, Özgöçmen, Armağan, & Semerci Sevimli 2022). ISM1 has 14 invariant cysteine residues, of which the TRS1 structural domain contains 6 and the AMOP structural domain contains 8 (Ciccarelli et al. 2002; Osório et al. 2014). This suggests that ISM1 may be associated with apoptosis. A study by Wei Xiang et al. (Xiang et al. 2011) confirmed this and showed that ISM1 induces caspase-3 activation in a dose-dependent manner, a key step downstream in apoptosis pathway, resulting in EC apoptosis. This study found that organ-secreted ISM1 is both soluble in blood and immobilized in the extracellular matrix (ECM) of tissues. The free ISM in the blood recruits caspase-8 to the plasma membrane and activates it in an αvβ5 integrin-dependent manner, inducing EC apoptosis through an in vitro pathway. The immobilized ISM is an αvβ5 integrin agonist that promotes EC adhesion and survival (Zhang et al. 2011). This suggests that ISM may mediate multiple aspects of the apoptotic process. (Fig. 4).

ISM1 binds to avβ5 and causes apoptosis via caspase-3 after activation by recruitment. ISM1 binds to GRP78, enters ECs through endocytosis, interacts with ACC on the inner mitochondrial membrane, interferes with ADP/ATP exchange, leads to a decrease in ATP levels in the cytoplasm, and induces apoptosis; In addition, BC71 in the AMOP domain of ISM1 binds to GRP78 to activate p53 and caspase-8 signaling, causing apoptosis.

Previous studies have shown that the endoplasmic reticulum (ER) chaperone protein, GRP78, promotes proper protein folding (Angeles-Floriano et al. 2022). GRP78 is a chaperone protein of ER. During stress, GRP78 is highly expressed on the surface of lymphocytes, endothelial cells, and various cancer cells (Davidson et al. 2005; Fradet et al. 2012; C. Liu et al. 2003; Tsai et al. 2015). Chen Ming et al. (M. Chen, et al. 2014) observed that ISM1 can induce apoptosis and exert its anti-tumor properties by regulating the high expression of GRP78 on the surface of mouse melanoma cells, human kidney cancer cells, and human liver cancer cells. This study showed that soluble ISM1 binds to GRP78, enters ECs through lattice-protein-dependent endocytosis, and reaches the mitochondria via late intracellular bodies (M. Chen et al. 2014). It then interacts with ACC, a key transporter protein in the mitochondrial inner membrane, and interferes with adenosine diphosphate/ATP(ADP/ATP) exchange, blocking the transport of ATP from the mitochondria to the cytoplasm and causing a decrease in the level of ATP in the cytoplasm, leading to the induction of apoptosis (M. Chen et al. 2014) (Fig. 3).

Additionally, a novel cyclic peptide, BC71, present in the AMOP structural domain of ISM1 was shown to be a pro-apoptotic ligand for GRP78 on the cell surface, and their combination could activate apoptotic signals like p53 and caspase-8 in human umbilical vein endothelial cells to cause apoptosis in cancer cells (Kao et al. 2018). Mice injected with BC71 intravenously were found to have inhibited growth of 4T1 mammary carcinomas and apoptosis was promoted in a caspase-8-dependent manner, suggesting that different protein sequences of ISM1 may promote apoptosis in different ways (Kao et al. 2018).

Non-coding RNA (ncRNA) is an important functional regulatory molecule mediating cellular processes, including transcription, post-transcriptional modifications, signaling pathways, and chromatin remodeling (Anastasiadou et al. 2018). ncRNA is involved in a variety of physiological and pathological processes, including cancer cell growth, differentiation, metastasis, and immune cell development, and it has functions in the tumor microenvironment; it is considered to be an oncogenic driver and tumor suppressor in many types of cancer (Xue et al. 2022). Among the various ncRNAs that have been identified are microRNA (miRNA) and circular RNA (circRNA). Zheng et al. (H. Li et al. 2014) showed that ISM1 is a miR-1307-3p target gene and that high expression of miR-1307-3p significantly inhibited ISM1 expression. In colon adenocarcinoma cell lines, miR-1307-3p expression was decreased and ISM1 expression was increased, promoting cell proliferation and inhibiting apoptosis through the Wnt/β-catenin signaling pathway (H. Li et al. 2014). circRNAs are thought to be miRNA sponges that can reverse the inhibitory effects of miRNAs (Anastasiadou et al. 2018). In hepatocellular carcinoma cell lines, hsa_circ_0091570 regulates ISM1 expression by sponging miR-1307 (Y. G. Wang et al. 2019), suggesting that future studies on ncRNAs and ISM1 in cancer deserve further exploration.

Similarly, it has been reported that ISM1 overexpression in gastric cancer inhibits apoptosis and promotes cancer cell proliferation (H. Li et al. 2014; Wu et al. 2021; Zheng, Zheng, Lei, Xiang, & Chen, 2019). Regarding the dual role of ISM1 in vivo, it may be that GRP78, a factor downstream of ISM1, acts synergistically with αvβ5 integrin, but both are mediated through different signaling pathways without significant co-localization in tumors, suggesting that both function as ISM1-independent surface receptors (M. Chen et al. 2014). In addition, it is unclear whether the dual role of ISM1 is due to the difference in disease classes or cancer cell types, or due to downstream levels of targeting in the pathways, and further studies are needed in the future to elucidate this. Additionally, GRP78 is expressed in a variety of cancer cells, but its regulatory role in cancer is still unclear, which needs to be further explored.

#### Anti-angiogenesis

Angiogenesis is the development of new blood vessels from existing capillaries or post-capillary veins. The purpose of angiogenesis is to meet the metabolic needs of cells, supply oxygen and nutrients, and remove metabolic waste (Parmar and Apte 2021). Cancer cells also need oxygen and nutrients. Under physiological conditions, angiogenesis ceases after cellular needs are met; however, in cancer cells, pro-angiogenic factors are continuously expressed to induce angiogenesis in tumors (Parmar and Apte 2021; Rajabi and Mousa 2017). Therefore, anti-angiogenesis is an important approach in the treatment of malignant tumors (Ozel et al. 2022).

The AMOP structural domain is found in some cell adhesion molecules and splice variants of MUC4, and thus may be associated with adhesion. In addition, the AMOP domain of ISM1 contains "RKD", which inhibits platelet function and interacts with αvβ5, suggesting that ISM1 may be associated with cell adhesion and angiogenesis (Zhang et al. 2011). Xiang W et al. (Xiang et al. 2011) compared the formation of capillary network after binding of full-length ISM1 protein, ISM1-TSR (TSR segment of ISM1), ISM1-C (AMOP segment at the C-terminus of ISM1), and ISM1-N (TSR segment + AMOP segment at the C-terminus of ISM1) to αvβ5 integrins on the surface of EC; they showed that ISM1 and ISM-C inhibited capillary angiogenesis in EC in a dose- and time-dependent manner and may affect cell migration, adhesion, and apoptosis. In vitro, ISM1 and ISM1-C could act as anti-angiogenic agents by interfering with early capillary angiogenesis (Xiang et al. 2011). In vivo, a mixture of VEGF and bFGF could induce angiogenesis in mice, which was inhibited when 0.5 μM and 1 μM of ISM1 were added, but not when ISM1-C was added under the same conditions, suggesting that there may be molecules in the organism that inhibit ISM1-C expression (Xiang, et al. 2011). Stable overexpression of ISM1 in B16 melanoma inhibited tumor growth in mice by suppressing tumor angiogenesis, and knockdown of ISM1 in zebrafish embryos caused abnormal formation of trunk intersegmental vessels. Moreover, administration of recombinant ISM1 could inhibit glioma growth by anti-angiogenesis with no significant side effects, which further proved the anti-angiogenic ability of ISM1(Xiang et al. 2011; Yuan et al. 2012). This suggests that ISM1 may be an endogenous inhibitor of angiogenesis, which could provide a direction for future tumor therapy, but the anti-angiogenic effect of ISM1 in other types of tumors remains to be explored.

#### ISM1 and immunization

The immune system has an important role in maintaining homeostasis in the body and consists mainly of innate and adaptive immunity. Adaptive immunity consists mainly of T lymphocytes which are involved in cellular immunity and B cells which are involved in humoral immunity. The body's immune cells, such as T cells and NK cells, play an immune role by destroying cancer cells in the body through the cancer-immune cycle (D. S. Chen and Mellman 2013; Peterson and Barry 2020). Valle-Rios et al. (Valle-Rios et al. 2014) showed that ISM1 was expressed in human oral mucosa, trachea, duodenum, mammary gland containing skin or mucosal tissues, NK cells and NKT cells in mouse lung, and CD4^+^ T lymphocytes in humans and mice. Interestingly, ISM1 was not expressed at the same level in different subpopulations of CD4^+^ polarization. ISM1 is overexpressed in Th17 cells, while ISM1 expression is low in regulatory T cells (Treg) and absent in Th1 and Th2 subpopulations, suggesting that ISM1 may be involved in the biological functions of NK, NKT, Th17, and other cells, and thus play a role in immunity(Valle-Rios et al. 2014).

Wu Y et al. found that ISM1 was highly associated with classical immune signaling such as TGF-β, IL-6/JAK/STAT3, IFN-γ, TNF-α/NF-κB, and IL-2/STAT5 by colorectal cancer gene enrichment analysis, and that most of these signaling pathways had effects on Treg cell infiltration, programmed death-ligand 1 (PD-L1) stability, and CD8^+^ T cell depletion. IL-6 plays a key role in the body's inflammatory response and tumor immunity (Hirano 2021; Johnson et al. 2018). Inflammation and cancer are linked through relevant external environments with intrinsic pathways, and STAT3 is a major intrinsic pathway linking inflammation and cancer. Sustained activation of IL-6/JAK/STAT3 signaling is an important factor in the development of cancer induced by inflammation (Yu et al. 2009). TNF-α/NF-κB and IL-6/JAK/STAT3 are common markers of Epstein–Barr virus-associated epithelial carcinoma, and ISM1 affects both pathways. Given that, ISM1 may also act on the above pathways to affect Epstein–Barr virus-associated epithelial carcinoma (Heawchaiyaphum et al. 2021). IL-2 is a pleiotropic cytokine that is phosphorylated upon binding to the cognate receptor IL-2Rβ and subsequently activates STAT5 signaling, acting primarily on Treg cells and CD8^+^ T cells (Y. Liu et al. 2021; MacDonald et al. 2021; Shi et al. 2018). Treg cells are characterized by the expression of CD4, CD25, forkhead transcription factor 3 (Foxp3), and they play a role in the prevention of autoimmune diseases, infectious diseases, and tumors (B. J. Chen, Zhao, Zhang, Zheng, and Wu 2022; Rajendeeran and Tenbrock 2021). IL-2/STAT5 has an important role in the differentiation and homeostasis of Treg and CD8^+^ T cells that are the key effector cells of tumor immunity (B. J. Chen et al. 2022; Frantz et al. 2022; Kolios et al. 2021; Rajendeeran and Tenbrock 2021). IL-2 promotes CD8^+^ T cell recognition and clearance of malignant cells for cancer immunotherapy in the early tumor stage, but sustained high levels of IL-2 in the tumor microenvironment induce CD8^+^ T cell depletion and dysfunction of T cells thus suppressing anti-tumor immunity (Y. Liu et al. 2021; MacDonald et al. 2021). ISM1 is also associated with T-cell depletion markers such as programmed cell death protein 1 (PD-1), lymphocyte activation gene-3 (LAG3), T-cell immunoglobulin and mucin-domain containing 3 (TIM-3), and cytotoxic T lymphocyte-associated protein-4 (CTLA-4), again validating that ISM1 may suppress the immune response of the body (Wu et al. 2021). In addition, 71% of the genes in the cancer-related signaling pathway epithelial-mesenchymal transition (EMT) is associated with ISM1, while EMT also induces Treg cells and dendritic cells and regulates immune responses (Kudo-Saito et al. 2009; Wu et al. 2021).

The expression of ISM1 was significantly induced in the liver, kidney, and spleen of zebrafish after 24 h and 48 h by Grass carp reovirus (GCRV). The recombinant ISM1 could induce interferon gene and interferon-induced antiviral protein expression through TANK binding kinase 1 (TBK1), interferon regulatory factor 3 (IRF-3), interferon-γ (IFN-γ) antiviral pathway, thus reducing GCRV-induced epithelioma papulosum cyprini cells (EPC) lesions and promoting antiviral immune response (C. Li et al. 2021).

Therefore, ISM1 seems to have a dual role in immunity: suppressing the body's immune response through the pro-inflammatory classical pathway; promoting the body's immune response through type I interferon. This dual mechanism of action remains to be explored. Although fish, mouse, and human ISM1 genes have high homology, the antiviral activity of ISM1 via TANK- IRF3-IFN-γ has only been experimented in zebrafish, and further studies are needed to confirm whether it has the same effect in other species. There are few studies on the role of ISM1 in inflammation and immune response in the body, and there are insufficient reports on the inhibition of tumorigenesis and metastasis via immune or inflammatory pathways, which may be a gap in the literature that is to be addressed in the future. In addition, ISM1 is associated with several classical inflammatory pathways, and obesity is highly associated with long-term chronic inflammation in the body, but whether ISM1 can directly affect obesity-related inflammation through these inflammatory pathways is still unclear and also deserves attention in future studies.

## Conclusion and outlook

In terms of structure, the high homology of ISM1 protein sequence and structural domains observed by bioinformatics suggests that ISM1 may play similar roles in different animals. ISM1 is dynamically expressed in various tissues and in different locations at different growth stages, suggesting that ISM1 may be closely related to the growth and development, as well as the functions, of these tissues and organs. Subsequent studies also confirmed its importance in growth and development, metabolism, and regulation of cancer. ISM1 is known to have two receptors, GRP78 and αvβ5, but it is not known whether there is synergy. ISM1 promotes glucose uptake via PI3K-AKT in mature adipocytes, but potential specific receptors have not been identified, and whether they have the same role in different stages of adipocytes is yet to be understood. ISM1 is closely connected with various inflammatory pathways, and whether it plays a role in the immunity of the body through these pathways also needs to be explored. Multiple forms of exercise play a role in metabolic diseases, and it is unknown whether exercise has an effect on ISM1, thus improving metabolic diseases. Although much exploration is still needed regarding ISM1 research, its roles in hematopoiesis, tumor immunity, diabetes, and nonalcoholic fatty liver disease have thus far been revealed, indicating the great therapeutic potential of ISM1. In addition, because there are few studies on the mechanism of ISM1, the various functions of ISM1 are not fully described and need to be further explored in the future.
